# Rediscovery of the Tubulin β‐4A p.Arg2Gly Variant in Whispering Dysphonia: A Report from Austria

**DOI:** 10.1002/mds.30227

**Published:** 2025-05-26

**Authors:** Omar Keritam, Susann Badmann, Maureen Jacob, Philip Harrer, Christine Klein, Annabella Kurz, Hakan Cetin, Michael Zech

**Affiliations:** ^1^ Department of Neurology Medical University of Vienna Vienna Austria; ^2^ Comprehensive Center for Clinical Neurosciences and Mental Health Medical University of Vienna Vienna Austria; ^3^ Institute of Human Genetics, Technical University of Munich, School of Medicine and Health Munich Germany; ^4^ Institute of Neurogenomics, Helmholtz Zentrum München Munich Germany; ^5^ Institute of Neurogenetics, University of Lübeck Lübeck Germany; ^6^ Department of Neurology University Hospital Schleswig‐Holstein Luebeck Germany; ^7^ Department of Otorhinolaryngology, Division of Phoniatrics‐Logopedics Medical University of Vienna Vienna Austria; ^8^ Institute for Advanced Study, Technical University of Munich Garching Germany

**Keywords:** dystonia, tubulin β‐4A, TUBB4A, laryngeal involvement, dysphonia

Whispering dysphonia (originally known as DYT4 dystonia) was characterized clinically in the 1980s in a single, large, multigenerational British‐descent family who immigrated to Australia.^1^ Affected individuals exhibited spasmodic dysphonia as the main phenotypic sign, variably accompanied by more widespread dystonic involvement.^1^ In 2013, two back‐to‐back publications reported a heterozygous variant involving the substitution of a single nucleotide in exon 1 of the tubulin β‐4A gene *TUBB4A* (c.4C>G, encoding p.Arg2Gly) as the cause of the family's condition (henceforth termed DYT‐*TUBB4A*).^2^, ^3^ Moreover, independent work implicated a broad set of other *TUBB4A* missense variants in hypomyelinating leukodystrophy‐6 (HLD6), a complex disorder featuring developmental delay, cognitive decline, neuroanatomical changes, and movement abnormalities including spastic tetraplegia and dystonia.^4^ Although a handful of additional families with DYT‐*TUBB4A* have been subsequently described,^5^, ^6^, ^7^ the original c.4C>G variant has never been found again. The clinical presentation associated with c.4C>G outside the original pedigree remained unknown. We encountered a 40‐year‐old woman who presented with progressive articulation impairment. At age 30 years, she began to develop a strangulated voice. Her speech became increasingly hoarse. She was unable to coordinate speech sounds and involuntarily whispered. In the years that followed, gradual deterioration occurred, resulting in longer episodes of complete inability to communicate verbally. There was no family history of similar symptoms or other neurological diseases. Examination revealed severe dysphonia with intermittent aphonia (Video 1), hyperreflexia in the extremities, and bilateral ankle clonus. She had no evidence of other dystonic manifestations and was cognitively intact. A prolonged latency of motor‐evoked potential responses was recorded, which was interpreted as an altered integrity of the pyramidal system. Magnetic resonance imaging studies of the brain and spine were normal. Continuous spasms of intrinsic muscles of the larynx were seen on laryngeal endoscopy. Whole‐genome sequencing detected the *TUBB4A* c.4C>G variant in a heterozygous state. The variant was confirmed as pathogenic based on American College of Medical Genetics and Genomics (ACMG) criteria. The patient's parents were deceased, and testing for de novo occurrence of *TUBB4A* c.4C>G was not possible. Our patient was of Austrian origin and acknowledged no familial relationships to the UK or Australia,^1^ indicating that we had likely identified a second, independent instance of DYT‐*TUBB4A* linked to the original mutation.^2^, ^3^ Alternative amino acid exchanges resulting from alterations of *TUBB4A* codon 2, such as p.Arg2Gln and p.Arg2Trp, have been observed in patients with HLD6 (https://www.ncbi.nlm.nih.gov/clinvar/), highlighting that the particular c.4C>G (p.Arg2Gly) change that we re‐identified here seems to be specifically associated with the peculiar presentation of DYT‐*TUBB4A*. Despite the absence of extra‐laryngeal dystonia, our present patient confirms the characteristic phenomenology of c.4C>G‐related DYT‐*TUBB4A*, which demonstrates dysphonia as a core defining feature.^7^ Notably, prior working clinical diagnoses for our patient comprised various speech disorders including motor neuron diseases such as primary lateral sclerosis, emphasizing the importance of accurate clinical and genetic evaluation. We wish to raise awareness of DYT‐*TUBB4A* as a differential diagnosis of unexplained progressive motor speech disorders, across different populations worldwide.

**Video 1:** Our patient with the *TUBB4A* c.4C>G (p.Arg2Gly) variant presenting with severe speech impairment.

## Author Roles

(1) Research Project: A. Study Design and Concept, B. Data Acquisition, C. Data Analysis and Interpretation, D. Clinical Examination, E, Supervision; (2) Statistical Analysis: A. Design, B. Execution, C. Review and Critique; (3) Manuscript Preparation: A. Writing of the First Draft, B. Revision of Manuscript for Critical Intellectual Content.

O.K.: 1B, 1C, 3B.

S.B.: 1C, 3B.

M.J.: 1C, 3B.

P.H.: 1C, 3B.

C.K.: 1C, 3B.

A.K.: 1C, 3B.

H.C.: 1B, 1C, 1D, 3B.

M.Z.: 1A, 1C, 1E, 3A.

## Financial Disclosures of All Authors (for the Preceding 12 Months)

C.K. reports consultancies from Centogene and Takeda; received honoraria from Desitin and Bial; and had advisory board participation for Retromer Therapeutics.

## Data Availability

The data that support the findings of this study are available on request from the corresponding author. The data are not publicly available due to privacy or ethical restrictions.
